# Advantages and Limitations of the Neonatal Immune System

**DOI:** 10.3389/fped.2020.00005

**Published:** 2020-01-28

**Authors:** George P. Tsafaras, Polyxeni Ntontsi, Georgina Xanthou

**Affiliations:** ^1^Cellular Immunology Lab, Center for Basic Research, Biomedical Research Foundation of the Academy of Athens, Athens, Greece; ^2^Second Respiratory Medicine Department, ‘Attikon’ University Hospital, National and Kapodistrian University of Athens, Medical School, Athens, Greece

**Keywords:** neonates, innate immunity, infections, sepsis, neurodevelopment, brain injury

## Abstract

During early post-natal life, neonates must adjust to the transition from the sheltered intra-uterine environment to the microbe-laden external world, wherein they encounter a constellation of antigens and the colonization by the microbiome. At this vulnerable stage, neonatal immune responses are considered immature and present significant differences to those of adults. Pertinent to innate immunity, functional and quantitative deficiencies in antigen-presenting cells and phagocytes are often documented. Exposure to environmental antigens and microbial colonization is associated with epigenetic immune cell reprogramming and activation of effector and regulatory mechanisms that ensure age-depended immune system maturation and prevention of tissue damage. Moreover, neonatal innate immune memory has emerged as a critical mechanism providing protection against infectious agents. Still, in neonates, inexperience to antigenic exposure, along with enhancement of tissue-protective immunosuppressive mechanisms are often associated with severe immunopathological conditions, including sepsis and neurodevelopmental disorders. Despite significant advances in the field, adequate vaccination in newborns is still in its infancy due to elemental restrictions associated also with defective immune responses. In this review, we provide an overview of neonatal innate immune cells, highlighting phenotypic and functional disparities with their adult counterparts. We also discuss the effects of epigenetic modifications and microbial colonization on the regulation of neonatal immunity. A recent update on mechanisms underlying dysregulated neonatal innate immunity and linked infectious and neurodevelopmental diseases is provided. Understanding of the mechanisms that augment innate immune responsiveness in neonates may facilitate the development of improved vaccination protocols that can protect against pathogens and organ damage.

## Introduction

During early life, newborns encounter a plethora of antigenic challenges derived from pathogens, commensals, and innocuous environmental antigens (1). In the face of an inexperienced adaptive immunity, innate immunity is critical for the survival of neonates. Still, deficits in innate immune cell functions, due to cell-intrinsic hyporesponsiveness concomitant with enhanced activation of immunosuppressive, tissue-protective mechanisms, render neonates vulnerable to infections, sepsis, brain damage and neurodevelopmental disorders (2–5). Rapidly-changing environmental and microbial exposures in the post-natal period, along with epigenetic reprogramming and innate immune memory, have also a major impact on neonatal immune responses (6). Despite significant scientific progress, the precise cellular and molecular mechanisms underlying defective neonatal innate immunity remain incompletely defined.

In this review, we present the most recent advances in the characterization of the phenotype and functions of neonatal innate immune cells, outlining the disparities with adult responses. Given that there is limited space to delve into the extensive series of animal studies, we focus on reports on human cells. In addition, we provide an overview of the effects of the microbiome, the metabolome, and epigenetics on the regulation of neonatal innate immunity. The immunological mechanisms underlying infections, brain injury, and neurodevelopmental disorders are also presented. Finally, we discuss future research directions that may boost neonatal host defense through targeting innate immune responses.

## Innate Immune Responses in Neonates

### Dendritic Cells

Human dendritic cells (DCs) mainly consist of two developmentally-distinct lineages; conventional (cDCs) that induce T cell activation and differentiation, and plasmacytoid DCs (pDCs), which produce type I interferons and mediate anti-viral responses (7). Neonatal cDCs are decreased in the peripheral blood and upon pathogen encounter [i.e., lipopolysaccharide (LPS) stimulation], secrete low levels of IL-12, leading to impaired T helper type (Th1) cell polarization (8). Decreased IL-12 synthesis by neonatal cDCs is associated with impaired chromatin remodeling in the gene promoter (9). Notably, neonatal cDCs secrete high levels of Th2 cell-associated cytokines, such as IL-4 and IL-13, which along with the anti-inflammatory cytokine IL-10, retain cDCs in an immature state (10). The expression of human leukocyte antigen (HLA)-DR and costimulatory molecules, such as CD40, CD80, and CD86, are also decreased in neonatal cDCs, reducing their antigen-presenting and T cell stimulatory functions (11). Defective production of IFN-β and IFN-inducible chemokine genes, including CXCL9, CXCL10, and CXCL11, by neonatal DCs is considered to result from decreased expression of IRF-3-dependent genes (9). Nevertheless, recent studies using cytometry by time of flight-assisted immunophenotypic analyses have shown that fetal and adult cDCs have similar expression of pattern recognition receptors (PRRs) and secrete equal amounts of GM-CSF, IL-6, CXCL8, and CCL4 following stimulation with several toll-like receptor (TLR) agonists [i.e., CL075, cytosine-phosphate-guanosine (CpG) oligodeoxynucleotides, polyinosinic-polycytidylic acid (PI:C), peptidoglycan (PGN)] *ex vivo* (12). These findings suggest that endogenous immunosuppressive and/or other factors may restrain neonatal cDC maturation and T cell stimulatory functions *in vivo*. Upon stimulation with TLR7 ligands, cytomegalovirus, herpes simplex virus-1 (HSV-1), or CpG, pDCs, also release decreased amounts of IFN-α, even though TLRs, HLA-DR, and costimulatory molecules are expressed at similar levels to their adult counterparts (13, 14).

### Monocytes and Macrophages

Monocytes play a key role in pathogen recognition and eradication through their phagocytic, antigen-presenting and cytokine-secreting abilities. Neonatal monocytes express decreased levels of HLA-DR and CD80, leading to impaired presentation of antigens, including pathogen-derived molecules (15). They are also characterized by reduced expression of membrane attack complex-1 and L-selectin, leading to decreased adhesion and infiltration to inflamed tissues (16). Interestingly, although stimulation with LPS enhances TLR4 expression, along with TNF-α, IL-6, and IL-10 secretion by neonatal macrophages, downstream TLR4 signaling pathways are impaired, as evidenced by reduced phosphorylation of NF-κβ-p65 and p38, and this may account for the overall decreased cytokine responses as compared to adult cells (17, 18). Neonatal monocytes also exhibit impaired activation of the nucleotide-binding domain and leucine-rich repeat containing protein 3 (NLRP3) inflammasome. In fact, following NLRP3 stimulation, low levels of caspase-1 lead to decreased pyroptosis and reduced secretion of active IL-1β (19). In contrast, neonatal monocytes express higher levels of the anti-apoptotic protein B-cell lymphoma 2 (Bcl-2), that prevents physiological termination of their responses and can exacerbate inflammatory processes (20). Additional studies have shown that macrophages produce high levels of migration inhibitory factor which enhances mitogen-activated protein kinase (MAPK) activation, and during sepsis, may lead to excessive cytokine release (14).

Compared to their adult counterparts, neonatal macrophages have increased cytoplasmic vacuolization and reduced expression of lipid residues, CD11b, CD14, and F4/80 (21). Still, upon pathogen encounter, they produce copious amounts of IL-6 and CCL2/3/4 (21). Given the inhibitory effects of IL-6 on neutrophil responses, the increased IL-6/TNF-α ratio in neonatal peripheral blood as described above, may account for the reduced neutrophilic migration to inflammatory tissue sites (22). Phagocytic responses of neonatal macrophages are similar to those of adults (23).

**Figure 1:**
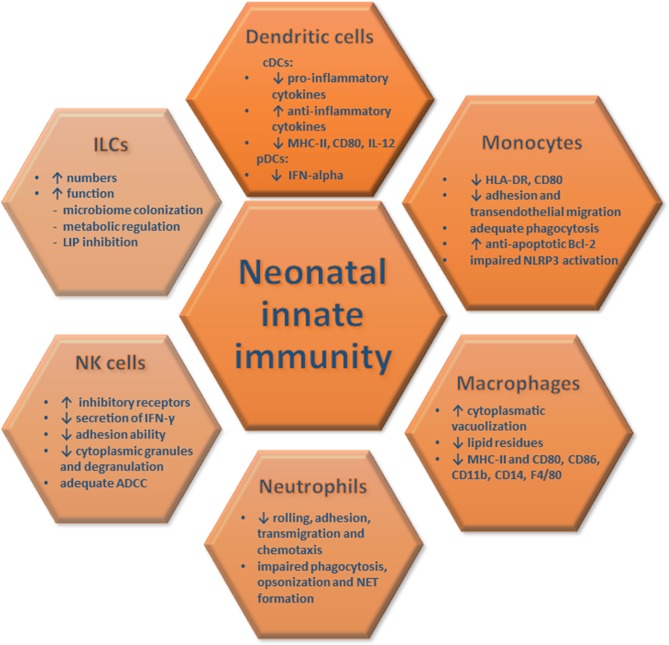
Innate immune responses in neonates. The quantitative and functional characteristics of neonatal innate immune responses are depicted and compared to those of adults. These features of innate immune cells render newborns vulnerable to severe infections and organ damage.

Overall, these studies highlight impaired antigen-presenting functions, cytokine secretion and T cell stimulatory abilities of neonatal DCs and monocytes/macrophages upon pathogen encounter, a phenomenon that renders neonates particularly vulnerable to infections (Figure 1). Still, it remains elusive whether the aforementioned defects are due to inherent immaturity of the neonatal innate immune system, the activation of endogenous immunosuppressive mechanisms or both, and future studies should further explore these clinically-relevant questions.

### Granulocytes

Neutrophils provide the first line of defense against pathogens through phagocytosis, release of toxic substances and generation of neutrophil extracellular traps (NETs). Neonatal neutrophils exhibit impaired rolling and adhesion capabilities, resulting from decreased expression of L-selectin, CD11b/CD18, and P-selectin glycoprotein ligand-1 (24, 25). They are also characterized by decreased calcium influx and intracellular calcium mobilization that affect actin polymerization, the microfilamentous cytoskeleton and chemotactic responses (26, 27). Moreover, decreased complement receptor 3 and nicotinamide adenine dinucleotide phosphate expression further reduce transendothelial migration and correlate with deficiency in opsonization and phagocytosis (25, 28, 29). NETosis is impaired compared to adult cells and lactoferrin, myeloperoxidase granule proteins and azurophilic protein are reduced in neonatal neutrophil extracts [(29); Figure 1]. Global proteomics analyses of cord and adult blood neutrophils validated the aforementioned findings showing downregulation of key proteins involved in proteasome, lysosome, and phagosome functions as well as in transendothelial migration (29). There is limited information pertinent to responses of other granulocytes. Animal studies have shown that neonatal basophils, through enhanced IL-4 production, trigger Th2-polarized responses and downregulate IL-12 release by cDCs (30).

### Natural Killer (NK) Cells

NK cells protect against pathogens through cytokine release and killing of infected cells. Expression of a complex repertoire of activating (CD94/NKG2C, killer immunoglobulin-like 1 receptor) and inhibitory receptors (CD94/NKG2A) enables NKs to sense infected and/or transformed cells (31, 32). NK numbers are increased during gestation, reaching their peak at birth, after which they decline reaching adult levels (31, 32). Inhibitory receptors are increased in neonatal NKs, endowing them with a cytokine-secreting rather than a cytotoxic profile. Nevertheless, the inhibitory receptor leukocyte immunoglobulin-like receptor-1, is reduced in newborn NKs (32). Cord blood NKs exhibit decreased degranulation abilities, as evidenced by reduced CD107c and lower production of perforin and granzymes, which result in poor cytotoxicity upon encounter of infected cells (31). In contrast, antibody-dependent cell-mediated cytotoxicity (ADCC) is not different to adults, as evidenced by equivalent Fcγ-RIII/CD16 levels [(32); Figure 1]. IFN-γ release is dampened in cord blood NKs mainly due to low IL-12 secretion by cDCs (33). NK adhesion is also impaired due to reduced CD62L/L-selectin and CD54/ICAM-1, whereas β2-integrin levels are similar to adult counterparts (34).

### Innate Lymphoid Cells (ILCs)

Innate Lymphoid Cells (ILCs) are characterized by the release of Th cell-associated cytokines and the absence of antigen-specific receptors. ILCs are classified into three subgroups (ILC1, ILC2, ILC3), based on their cytokine and transcription factor profile and represent essential drivers of early host responses during infection and injury (35). ILCs presence and functionality is increased during infancy, wherein ILCs contribute to lymphoid tissue formation and gut homeostasis by regulating microbiome installation and metabolic processes (36, 37). During intra-amniotic infection, ILCs migrate to the amniotic fluid and participate in tissue repair (36). Interestingly, neonatal mouse ILC3s inhibit lymphopenia-induced proliferation (LIP), a process that leads to aberrant T cell activation, tissue destruction and loss of T-cell receptor diversity (37). This creates a diverse repertoire of naive T cells that is essential for T cell homeostasis and induction of immunological tolerance (Figure 1). The human infant oral epithelium exhibits a predominance of ILC2s that produce Th2 cytokines, important for defense against extracellular bacteria and parasites (38).

## Factors Influencing Neonatal Innate Immunity

### Epigenetics

Epigenetic modifications, including acetylation, methylation, ubiquitination, phosphorylation and sumoylation on histones, and/or DNA, as well as, microRNA-mediated regulation of translation have a major impact on innate immunity. Studies conducted during the post-natal period have shown a decrease of the histone mark H3K4me1 in monocytes, concomitant with a gradual increase of the H3K4me3 mark, mostly at promoter regions (39). The decreased H3K4me3 abundance early-on at pro-inflammatory genes, including CCR2, IL-1β, and TNF-α, is associated with reduced expression (39). In fact, the increased ratio H3K4me1/H3K4me3 may also underlie the impaired antigen-presenting functions of neonatal monocytes (39). Interestingly, low levels of H3K4me3 at genes encoding aerobic glycolysis pathways are also observed in neonatal monocytes (40). Considering that a metabolic shift from oxidative phosphorylation to glycolysis and/or lipid metabolism is pivotal for the induction of immune response genes in macrophages, these observations have important ramifications for the increased susceptibility of neonates to infections. Another study showed that monocytes derived from newborns born to obese mothers display reduced LPS responsiveness, associated with altered cytokine promoter methylation (41). In contrast, CpG methylation patterns in cord blood NKs did not differ from their adult counterparts (42). Pertinent to microRNAs, cord blood monocytes have increased miR146a that represses TLR4 signaling and pro-inflammatory cytokine secretion (43).

### Microbiome

The microbiome plays a catalytic role in the maturation of neonatal immune responses. In fact, only during juvenility, wherein the microbiome shifts to the adult composition, the establishment of a fully-operating immune system occurs (44). Microbial colonization was considered to occur during and following delivery (44). However, bacterial DNA was recently detected in the placental tissue and amniotic fluid, suggesting that initiation of microbiome colonization occurs prenatally (41, 45). Several prenatal and perinatal factors, including the mode of delivery, antibiotics consumption and diet, shape the microbial community, and consequently, the maturation of neonatal immunity. The neonatal microbiome exhibits lower diversity, compared to adults (44). Microbiota mainly consisting of *Bacteroidetes, Firmicutes, Proteobacteria, Actinobacteria, Enterobacteriaceae*, and *Bifidobacterium* are essential for the generation of an anaerobic environment in the neonatal intestine and compete with pathogens for nutrients, pH, adhesion sites and production of metabolites (44).

Experimental studies using germ-free mice demonstrated that the microbiome affects macrophage development and polarization, granulocyte numbers and haematopoiesis during early life (46, 47). For example, the presence of *Lactobacillus* in the maternal vagina correlates with IL-12 levels in neonatal cord blood, while an inverse correlation exists between *Bacteroides fragilis* and LPS-induced CCL4 and IL-6 production by mononuclear cells (48). Decreased presence of the *Bacteroidetes* phylum is also associated with lower plasma CXCL10 and CXCL11 levels in infants (48).

Collectively, the aforementioned studies suggest that epigenetic alterations and the microbiome composition greatly affect neonatal innate immune responses; however, the precise immunological and molecular mechanisms involved remained incompletely explored. Further animal studies and *ex vivo* analyses of human clinical samples are needed to delineate these intricate interactions and understand how they can be harnessed for the enhancement of protective immunity in neonates.

### Metabolome

The past years, there is an increasing interest in the analysis of the metabolome of biological fluids, including the amniotic fluid, the cord and the peripheral blood, the saliva, and the urine in newborns (49). Neonates exhibit a constantly-changing metabolomic profile that correlates with alterations in their environment, diet and the microbiome. For example, glycine is an essential amino acid that participates in glutathione synthesis and protects against oxidative stress, promotes the formation of purines, hemes, collagen and elastin, and enhances neurotransmission (49). Glycine is increased in neonates, and especially in preterms and/or newborns with intrauterine growth restriction, compared to adults, a process possibly associated with their enhanced metabolic demands (50). Choline, a precursor of lipoproteins and phospholipids involved in neurotransmission, is increased in neonates with cerebral damage and sepsis and contributes to metabolic imbalance (51–53). In contrast, reduced choline levels are detected in preterm and low birth-weight neonates and associated with decreased survival and impaired energy demands (54).

The interdependency of the microbiome, diet and the metabolome is exemplified by the colonization of the neonatal intestine early-on during development by the *Bifidobacterium* species that plays a key role in the generation of oligosaccharides through breastfeeding (55). Moreover, recent studies have shown that the metabolome is significantly altered in germ-free mice (56), while the administration of probiotics and prebiotics affect metabolite composition (57, 58). The levels of gluconate, a fundamental metabolite provided through glucose oxidation or products of *Enterococcus faecalis* and *Escherichia coli*, are enhanced in preterm neonates with necrotizing enterocolitis (NEC), which are also characterized by an altered microbiome composition (59, 60). Considering that the metabolomics field is still in its infancy, further studies are required to delineate its precise role in orchestrating innate immune responses in neonates. Still, the generation of a metabolome “map” is expected to significantly enhance the identification of new disease biomarkers, provide crucial opportunities for early diagnosis and facilitate the understanding of unknown aspects of the neonatal physiology.

## Dysregulation of Neonatal Innate Immunity

### Infections

Dysregulated innate immune responses render newborns susceptible to severe infections. Neonatal sepsis occurs predominantly in response to respiratory tract infections and meningitis, and represents a major cause of morbidity and mortality in the neonatal period, especially in very low birth-weight preterm infants (61). *In utero* infection is a significant risk factor for the development of early onset (EOS) sepsis (61, 62). EOS and late-onset sepsis (LOS) are characterized by differences in the time of infection and the way of transmission (61, 62). *Group B Streptococci* and *Escherichia coli* are predominantly involved in EOS, while *Coagulase negative Staphylococci* is observed in LOS (63). Viral infections, including HSV, enteroviruses, and parechoviruses, are also implicated in EOS (61).

**Figure 2:**
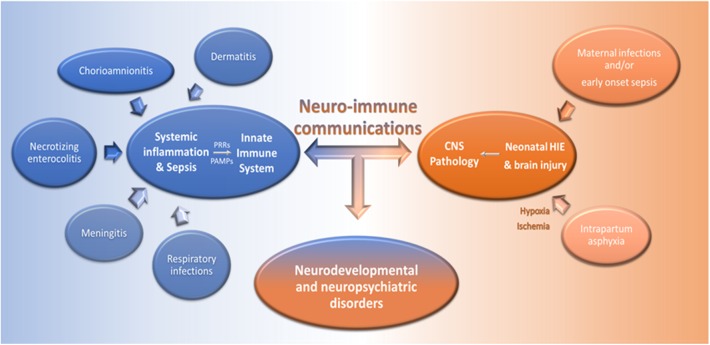
Dysregulated neuro-immune communication underlies the pathology of infections and CNS disorders in neonates. Infections, such as chorioamnionitis, dermatitis, meningitis, necrotizing enterocolitis, and respiratory infections, can lead to systemic inflammation and severe sepsis in newborns. Pathogen associated molecular patterns (PAMPs), generated during infections, are recognized via PRRs by innate immune cells and this induces cell activation and the production of pro-inflammatory mediators in the periphery and the CNS. On the other hand, maternal infections during pregnancy and/or EOS, and intrapartum asphyxia can induce brain injury and HIE, respectively, and further activate innate immune responses. Dysregulated neuro-immune communications underlie the pathogenesis of systemic infections and brain damage in neonates and may lead to neurodevelopmental and neuropsychiatric disorders.

Accumulating evidence illuminates critical defects of neonatal innate immunity during sepsis. Septic neonates present deficiencies in the recognition of pathogen products, including LPS, and in TNF-α, IFN-γ, IL-12 release that stem on TLR (especially TLR4) defects and reduced intracellular signaling by myeloid differentiation primary response protein 88 and MAPK p38 [(43); Figure 2 and Supplemental Table 1]. Moreover, preterms exhibit decreased LPS-induced TNF-α release, while cDCs secrete reduced amounts of IL-12, compared to non-septic preterms (5). In contrast, circulating IL-10 concentrations are elevated during EOS and LOS, enhancing an immunosuppressive milieu and limiting the ability of innate immune cells to eliminate pathogens (64). High levels of plasma adenosine trigger intracellular accumulation of immunosuppressive cAMP and further inhibit LPS-induced cytokine production (65). C9 levels are approximately 50% of their adult counterparts and linked to deficient opsonization and bacterial killing (66). Reduced levels of opsonins, mannose-binding lectin, bactericidal permeability-increasing protein and human neutrophil peptide also correlate with a high incidence of neonatal sepsis (5). Pertinent to cellular responses, macrophages produce decreased levels of reactive nitrogen intermediates (67), while monocytes express lower HLA-DR (11). Functional and quantitative deficiencies of neonatal neutrophils also increase the risk of development and dissemination of bacterial infections (65). For example, deficient NET formation and NET-mediated killing is observed during sepsis (68). Moreover, decreased circulating levels of TNF-α, IFN-γ, IFN-α, and IL-12 during neonatal sepsis impair NK cell-mediated cytotoxicity [(62, 69); Figure 2 and Supplemental Table 1].

Strikingly, neonates can also exhibit exaggerated innate immune responses that may lead to severe organ damage. Indeed, systemic inflammation during neonatal infections is closely linked to brain injury and neurological impairments (70, 71). The levels of IL-6 (72), CXCL8 (73), and IFN-γ (72) are elevated in the circulation of septic neonates and correlate with disease severity, whereas TNF-α and IL-1β production is more variable (74, 75). In chorioamnionitis, occurring as a result of intra-uterine infection and/or sterile inflammation, invasion of microorganisms or release of stress factors (i.e., danger signals and alarmins) in the amniotic cavity augments IL-1β, IL-6,TNF-α, CXCL8, and CXCL6 secretion and activates NLRP3 signaling (76, 77). This enhanced chemotactic gradient stimulates neutrophilic infiltration from the decidua into the chorioamniotic membranes and may lead to necrotizing chorioamnionitis, premature rupture of membranes, NEC, bronchopulmonary dysplasia, and periventricular leukomalacia [(78); Figure 2 and Supplemental Table 1]. Aberrant macrophage activation is also implicated in this exaggerated response (77). In the context of preterm labor induced by chorioamnionitis, fetal inflammatory response syndrome, severe dermatitis, and pneumonitis may be developed (78). In contrast, chorioamnionitis is associated with a reduced risk of LOS, possibly due to the establishment of innate immune memory that enhances protective immunity upon subsequent pathogen exposure (78).

### Neurodevelopmental Disorders

Several molecules involved in innate immunity, including cytokines, the complement cascade and adhesion molecules, are expressed in the healthy brain wherein they play essential roles in neurogenesis, migration, differentiation, synapse formation, and plasticity (79, 80). Still, dysregulated expression of these factors during early life can have deleterious consequences on brain development and function.

Intrapartum asphyxia causes hypoxia-ischemia (HI) which induces long-term neurological disorders, including cerebral palsy, visual impairment, seizures, epilepsy, mental retardation, and learning disabilities (81). Neonatal hypoxic-ischemic encephalopathy (HIE) is characterized by extensive neuronal cell death (81). Danger signals derived from dead and/or apoptotic neurons, including IL-33, high-mobility group protein B1 and ATP, are recognized by microglia, astrocytes and perivascular macrophages and induce their activation and release of pro-inflammatory cytokines, chemokines, nitric oxide synthase, ROS and excitatory amino acids (82). Furthermore, TNF-α, IL-6, IL-1β, and IL-12 release increase the blood-brain barrier permeability, augmenting the infiltration of DCs, and monocytes/macrophages into the brain (2). IL-6R, IL-1βR, and IL-18R activation in brain endothelial cells also increases production of TNF-α, activator protein 1 and prostaglandins (82). Altogether, these factors alter neuronal excitability and neurotransmitter function as they induce an imbalance in gamma-aminobutyric acid and glutamate, promoting long-term defects on synapse formation and neurogenesis (73, 74, 77). Newborns with HIE have elevated expression of TNF-α and IL-1β in the serum and cerebrospinal fluid (CSF), which correlate with brain injury severity (83). CXCL8 and IL-6 are also increased in the blood and CSF during perinatal asphyxia and correlate with HIE severity and neurological outcome (83–85). IL-10 is higher in the serum of asphyxiated neonates, while enhanced neutrophilic infiltration during the first 96 h is associated with poor neurodevelopmental outcomes [(86, 87); Figure 2 and Supplemental Table 1].

Autism spectrum disorders (ASD), Alzheimer's disease, major depression and schizophrenia, are considered as closely linked to three interrelated mechanisms; (a) dysregulated neuro-immune communication, (b) dysbiosis of the gut microbiome, and (c) early-life infections (3, 4, 88, 89). Animal studies have demonstrated that maternal viral infections during gestation, along with aberrant activation of innate immunity, have a long-lasting impact on offspring brain development and anxiety behavior, often in a sex-dependent manner, due to the activation of the hypothalamic-pituitary-adrenal axis (90). Children with ASD exhibit impaired NK cytotoxicity (91) and elevated production of TNF-α, IL-2, CXCL8, and IL-6 in the CSF (92). CCL2 and CCL5 are also increased in the brain of autistic children and associated with microglial activation and behavioral changes (92, 93). Increased IL-1β and IL-4 are associated with severe ASD (94), while IL-10 and TGF-β are decreased in the circulation (95). In contrast, neuroprotective factors, including brain-derived neurotrophic factor and Bcl-2, are decreased in ASD children [(96, 97); Figure 2 and Supplemental Table 1]. Interestingly, newborns with sepsis exhibit an increased risk of brain injury compared to those with chorioamnionitis, suggesting that similar to its protective effects on subsequent infections, chorioamnionitis preconditions the developing brain against subsequent injury induced by HI (98).

## Discussion

It is becoming increasingly clear that the development of immune responses in neonates is compromised not only as a result of immaturity but also as an attempt to maintain tolerance to innocuous and commensal antigens and prevent tissue damage. Still, once neonatal immune responses are activated, their magnitude is often such that they can cause severe immune pathology and morbidity, especially during infections and HI (Figure 2 and Supplemental Table 1). Hence, the provision of broadly-active innate immune stimuli during early life may protect against the detrimental consequences of uncontrolled inflammation, concomitant with the establishment of protective immunity. Vaccine-induced immune responses in neonates are impaired in terms of quantity and quality and multiple boosters are required to promote immunological memory (99, 100). On the other hand, vaccination with Bacille Calmette-Guerin (BCG) decreases neonatal mortality caused by infections by promoting heterologous lymphocyte activation against antigenically diverse and unrelated pathogens and through enhancing innate immune memory (101). Still, apart from BCG, hepatitis B and oral poliovirus, there are no other vaccines given after birth, highlighting a critical unmet need for this vulnerable young population (99, 102). It becomes evident that future studies should focus on enhancing vaccine effectiveness through stimulation of innate immune responses, for example, via administration of synthetic TLR8 agonists, including imidazoquinolines and single-stranded RNAs (99, 100). Alternatively, targeting TLR-independent pathways and bypassing immunosuppressive networks can also boost protective immunity without compromising crucial homeostatic functions.

Intricate interactions between the microbiome and the nervous and the immune systems affect neonatal responses during infections and brain injury. As such, alterations in the microbial composition, for example through dietary supplementation, may modulate key pathways of neuroimmune communication and enhance regulatory mechanisms that prevent neuronal damage. Interventions, including corticosteroids, melatonin, erythropoietin, anti-TNF-α antibodies, and IL-1rα administration, in clinical use for other diseases, may be also utilized for the control of neurological disorders in susceptible neonates (103–107). Finally, advances in systems-based approaches, including high-resolution genomic, proteomic, and metabolomic technologies are expected to provide new insight into the mechanisms underlying gut, brain and immune system interactions, and guide the development of more accurate diagnostic tests and therapeutic interventions.

## Author Contributions

GT and PN searched the literature and wrote the manuscript. GT designed the figures. GX contributed to critical revision of the article, suggested additional references, and wrote the manuscript. All authors read and approved the final manuscript.

### Conflict of Interest

The authors declare that the research was conducted in the absence of any commercial or financial relationships that could be construed as a potential conflict of interest.

## Abbreviations

DCs: dendritic cells
cDCs: conventional dendritic cells
pDCs: plasmacytoid dendritic cells
LPS: lipopolysaccharide
Th1: T helper type 1
PRRs: pattern recognition receptors
TLR: Toll-like receptor
CpG: cytosine-phosphate-guanosine
PI:C: polyinosinic-polycytidylic acid
PGN: peptidoglycan
HSV: herpes simplex virus
HLA-DR: human leukocyte antigen-DR isotype
BCL-2: B-cell lymphoma 2
MAPK: mitogen-activated protein kinase
NLRP3: nucleotide-binding domain and leucine-rich repeat containing protein 3
NETs: neutrophil extracellular traps
NK: natural killer
ILCs: Innate Lymphoid Cells
NEC: necrotizing enterocolitis
EOS: early-onset sepsis
LOS: late-onset sepsis
HI: hypoxia-ischemia
HIE: hypoxic-ischemic encephalopathy
CSF: cerebrospinal fluid
ASD: autism spectrum disorders.
