# Supplementary material for: Advantages and Limitations of the Neonatal Immune System
Source: Front Pediatr. 2020 Jan 28;8:5. doi: 10.3389/fped.2020.00005 (PMC6997472; doi:10.3389/fped.2020.00005)
Supplement: Supplementary file 1 [file Data_Sheet_1.PDF]

Supplemental Table 1: Alterations in innate immune responses during neonatal infections and neurodevelopmental disorders

**Sepsis/Infections**	**Hypoxic-ischemic encephalopathy**
↓ PAMPs recognition<br>↓ NK cytotoxicity	↑ neuronal cell death<br>↑ IL-33, high-mobility group protein B1 and ATP
↓ NET formation and killing by neutrophils<br>↓ macrophage activation	↑ microglial and astrocyte activation
↑ IL-10, adenosine<br>↓ TNF-α, IL-12, IFN-γ	↑ TNF-α, IL-6, IL-1β, IL-12, CXCL8, IL-10<br>↑ ROS, NOs, prostaglandins, AP-1
↓ mannose-binding lectin, opsonins, anti-microbial peptides, complement (C9) levels	↑ infiltration of neutrophils and peripheral macrophages into the brain
**Chorioamnionitis**	**Neurodevelopmental disorders**
↑ IL-6, CXCL8, CXCL6, IL-1β, TNF-α	↑ TNF-α, IL-2, CXCL8, IL-6, CCL2, CCL5, IL-1β, IL-4
↑ NLRP3 activation	↓ IL-10 and TGF-β
↑ neutrophilic and macrophage infiltration	↓ neuroprotective factors (BDNF, Bcl-2)
